# A Real-Time Wearable System for Monitoring Vital Signs of COVID-19 Patients in a Hospital Setting

**DOI:** 10.3389/fdgth.2021.630273

**Published:** 2021-09-07

**Authors:** Mauro D. Santos, Cristian Roman, Marco A. F. Pimentel, Sarah Vollam, Carlos Areia, Louise Young, Peter Watkinson, Lionel Tarassenko

**Affiliations:** ^1^Department of Engineering Science, Institute of Biomedical Engineering, University of Oxford, Oxford, United Kingdom; ^2^Critical Care Research Group, Nuffield Department of Clinical Neurosciences, University of Oxford, Oxford, United Kingdom

**Keywords:** electronic track & trigger, COVID-19, wearable devices, isolation wards, vital signs, continuous monitoring, e-obs, remote patient monitoring

## Abstract

The challenges presented by the Coronavirus disease 2019 (COVID-19) pandemic to the National Health Service (NHS) in the United Kingdom (UK) led to a rapid adaptation of infection disease protocols in-hospital. In this paper we report on the optimisation of our wearable ambulatory monitoring system (AMS) to monitor COVID-19 patients on isolation wards. A wearable chest patch (VitalPatch®, VitalConnect, United States of America, USA) and finger-worn pulse oximeter (WristOx2® 3150, Nonin, USA) were used to estimate and transmit continuous Heart Rate (HR), Respiratory Rate (RR), and peripheral blood Oxygen Saturation (SpO_2_) data from ambulatory patients on these isolation wards to nurse bays remote from these patients, with a view to minimising the risk of infection for nursing staff. Our virtual High-Dependency Unit (vHDU) system used a secure web-based architecture and protocols (HTTPS and encrypted WebSockets) to transmit the vital-sign data in real time from wireless Android tablet devices, operating as patient data collection devices by the bedside in the isolation rooms, into the clinician dashboard interface available remotely *via* any modern web-browser. Fault-tolerant software strategies were used to reconnect the wearables automatically, avoiding the need for nurses to enter the isolation ward to re-set the patient monitoring equipment. The remote dashboard also displayed the vital-sign observations recorded by the nurses, using a separate electronic observation system, allowing them to review both sources of vital-sign data in one integrated chart. System usage was found to follow the trend of the number of local COVID-19 infections during the first wave of the pandemic in the UK (March to June 2020), with almost half of the patients on the isolation ward monitored with wearables during the peak of hospital admissions in the local area. Patients were monitored for a median of 31.5 [8.8, 75.4] hours, representing 88.1 [62.5, 94.5]% of the median time they were registered in the system. This indicates the system was being used in the isolation ward during this period. An updated version of the system has now also been used throughout the second and third waves of the pandemic in the UK.

## Introduction

The Coronavirus disease 2019 (COVID-19) was declared a global health emergency by the World Health Organisation (1) at the beginning of March 2020. In its early stages, this pandemic presented several challenges for in-hospital patient care in the United Kingdom (UK): the fear of nosocomial infections in clinical environments, the lack of knowledge about the dynamics of virus transmission and initial shortages of Personal Protective Equipment (PPE). These rapidly led to a reduction in hospital admissions for non-COVID-19 disease, e.g., for cancer treatment (2), and the need for rapid adaptation of existing infectious disease protocols across the hospital (3, 4).

While the most severely ill COVID-19 patients were admitted to the Intensive Care Unit (ICU), those who did not meet ICU admission criteria were placed under observation on isolation wards (5). Our research group, a collaboration between clinical staff and biomedical engineers, was tasked by hospital management in Oxford at the end of February 2020 with supplying them with the most appropriate vital-sign monitoring system for these isolation-ward patients. Six priority requirements for the system were established (by order of appearance in the manuscript):

1. Because COVID-19 is primarily a disease which affects the cardio-respiratory system, it was decided that the three most important physiological parameters to monitor, ideally on a continuous basis, should be peripheral blood Oxygen Saturation (SpO_2_), Respiratory Rate (RR), and Heart Rate (HR).
2. The patients should not be confined to bed but should ideally be ambulatory, an important factor in the recovery from respiratory disease, for those patients stepping down from Intensive Care.
3. The patients were to be remotely monitored within the hospital, in the sense that they would be in individual rooms on the infection wards, with the nursing staff caring for them situated in another location nearby (the “nurse bays”), on the same hospital floor.
4. Any additional continuous monitoring from wearables should be fully integrated with the periodic nurse observations of the full set of vital signs, comprising SpO_2_, RR, HR as well as Blood Pressure (BP), Temperature (Temp), level of consciousness and the corresponding Early Warning Score (EWS) (6), in use throughout UK hospitals. The totality of the patient information (continuous data from wearables and periodic nurse observations) should be made available on real-time displays on a central station in the nurse bays, away from the isolation ward.
5. The amount of contact between the infected patients and the nursing staff was to be minimised, which meant that the frequency of nurse observations, which required the use of PPE, could not be increased, even though it was already known that the COVID-19 disease could lead to *rapid* patient deterioration.
6. The system should work within the hospital cyber-security infrastructure, compliant with patient confidentiality standards.

It soon became clear to us that the wearable ambulatory monitoring system (AMS) which we had been developing to monitor high-risk patients continuously on general wards, and create a virtual High-Dependency Unit (vHDU), could be adapted to meet the above six requirements.

In the next section, we review state-of-the-art wearable ambulatory monitoring systems, before providing an overview of our vHDU system, based on commercial off-the-shelf components, indicating how we had previously assessed their wearability, accuracy, and reliability. We then describe how this system was optimised to ensure that it met all of the above six requirements and deployed to monitor patients infected with the COVID-19 virus during the first wave of the pandemic in the UK, from mid-March to June 2020. Finally, we discuss preliminary results of the system usage in the isolation ward during this period.

## Wearable Ambulatory Monitoring Systems

State-of-the-art wearable AMS present a combination of mechanical (e.g., accelerometer), physiological (e.g., Electrocardiogram, ECG), and biochemical sensors (e.g., glucose monitors) (7) and include adhesive patch, clothing, chest-strap, upper-arm band, wristband, and finger-worn monitors (8). ECG, HR, RR, SpO_2_, BP, Temp, and patient activity are the clinical parameters most commonly tracked by current AMS deployed for in-hospital monitoring (9).

The wireless technologies found in these wearable AMS range from Wi-Fi and Bluetooth-Low-Energy (BLE) to cellular and Radio-Frequency (RF) technologies. Data are usually transmitted from the wearable(s) to an intermediate wireless client (e.g., a tablet or smartphone, in a 1-to-1 routing configuration) and then to an intranet or cloud server *via* Wi-Fi routers. Alternatively, several wearables can transmit data to a single access point (N-to-1 routing configuration), which then relays them to servers *via* a wired network. Data from several patients are processed for display and are typically reviewed by clinicians on workstations or ward screens (often wall-mounted) and mobile applications, in parallel and in real-time.

Examples of certified AMS with published validation or feasibility studies in the hospital setting include (10):

- The Vista Solution™ (VitalConnect, United States of America, USA), which uses a disposable 5-day battery chest-patch to collect ECG, HR, RR, body Temp, and activity data into a client tablet *via* BLE (1-to-1 routing). The tablet, in turn, retransmits the data over Wi-Fi into the Vista Solution™ cloud platform. BP and SpO_2_ can also be collected *via* third-party BLE devices.
- The ViSi Mobile System (Sotera Wireless, USA), which uses one Wi-Fi enabled wrist-worn module, connecting to one finger-probe pulse oximeter (for SpO_2_), to one 3- or 5-lead ECG chest module (for ECG/HR/RR/skin Temp/activity) and to one upper-arm cuff module (for BP). The module transmits the data to an intranet server, *via* the hospital Wi-Fi routers (N-to-1 routing).
- The Sensium® System (Sensium Healthcare Ltd, UK), which uses a re-usable RF chest patch (disposable electrodes), connected to an axilla probe, to measure HR, RR and body Temp. A proprietary RF wireless router collects data from several patches (N-to-1 routing) and retransmits them to a central server *via* the hospital Wi-Fi (or wired connexion).

For all three examples, the centralised server/cloud data are then made available to remote clinicians *via* a web-, desktop application- or mobile app- based dashboards, on which it is possible to review numerical data such as HR, RR, BP, referred to also as “numerics” thereafter, waveforms such as the ECG, and alert/notifications. Other in-hospital AMS broadly follow similar architectures.

To date there have been no randomised controlled trials with wearable based AMS that show significant clinical benefit/cost-effectiveness, and clinical studies are still needed before these systems can be adopted in large-scale clinical practise (10).

## The Oxford Virtual HDU Ambulatory Monitoring System

### Background Work

When we originally set out to identify suitable wearable devices for our vHDU system, we focused on devices which also provide the raw waveforms, i.e., the Photoplethysmogram (PPG) and the ECG *via* wireless-transmission mode (e.g., BLE). The availability of waveforms, not only enables further biomedical signal processing research, but also allow clinical staff to review these waveforms on bedside monitors to confirm the correct application of the sensors to the patients.

In a first study, we assessed the wearability of a selection of commercially-available wearables for monitoring the vital signs of ambulatory patients (11). Our study, which used a prospective observational cohort design, was reviewed and approved by the Oxford University Research and Ethics Committee and Clinical Trials and Research Governance teams (R55430/RE003). Participants in the study were required to wear up to four different AMS for up to 72 h each to mimic in-hospital use.

Next, a clinical accuracy study was conducted, in which up to 33 healthy participants undertook six different motion tasks followed by an hypoxia exposure phase (study approved by the East of Scotland Research Ethics Service REC 2 (19/ES/0008), study number ISRCTN61535692). During exposure to hypoxia, participants were desaturated under controlled conditions to seven SpO_2_ targets: {100, 95, 90, 87, 85, 83, 80}%, controlled *via* an oxygen mask (overseen by an anaesthetist), whilst wearing four wearable pulse oximeters, one standard Philips MX450 monitor pulse oximeter, one adhesive chest patch, and were fitted with an arterial line. The protocol of this study can be found in Areia et al. (12).

The accuracy of the pulse oximeter devices was assessed by comparing their SpO_2_ and Pulse Rate (PR) estimates with simultaneous gold-standard arterial blood oxygen saturation (SaO_2_), measured from arterial blood gas sampled *via* the arterial line, and with ECG-derived HR, respectively (we make the assumption that PR and HR can be used interchangeably). The accuracy of the chest patch was evaluated by comparing its RR and HR estimates with simultaneous RR and HR estimates derived from the reference capnography and the ECG. Full results for both of these evaluations can be found in Santos et al. (13) and Morgado et al. (14), respectively.

As a result of our wearability and accuracy evaluations, the WristOx® 3150 OEM BLE (Nonin Medical Inc., USA) (15) finger-based pulse oximeter, named Nonin hereafter, and the VitalPatch® (VitalConnect, USA) (16) adhesive chest-patch were ultimately selected as our wearable devices. From the Nonin, we collect the PR, SpO_2_ (both at a sampling rate of 1 Hz) and near-infrared PPG waveform (at a sampling rate of 75 Hz). From the VitalPatch®, we collect the HR and RR (both at a sampling rate of 0.25 Hz), patient posture (e.g., standing, sitting, lying down, etc.), number of steps (at a sampling rate of 1 Hz) and the single-lead ECG and 3-axis accelerometer waveforms (at sampling rates of 125 and 62.5 Hz, respectively). Both devices compute signal-quality indices and display battery status. BLE technology allows these wearable devices to stream the combination of HR, RR, and SpO_2_ data from active patients into a BLE-enabled tablet, continuously for ~48 h and 5 days, respectively (meeting the first and second requirements from section Introduction).

**Figure 1:**
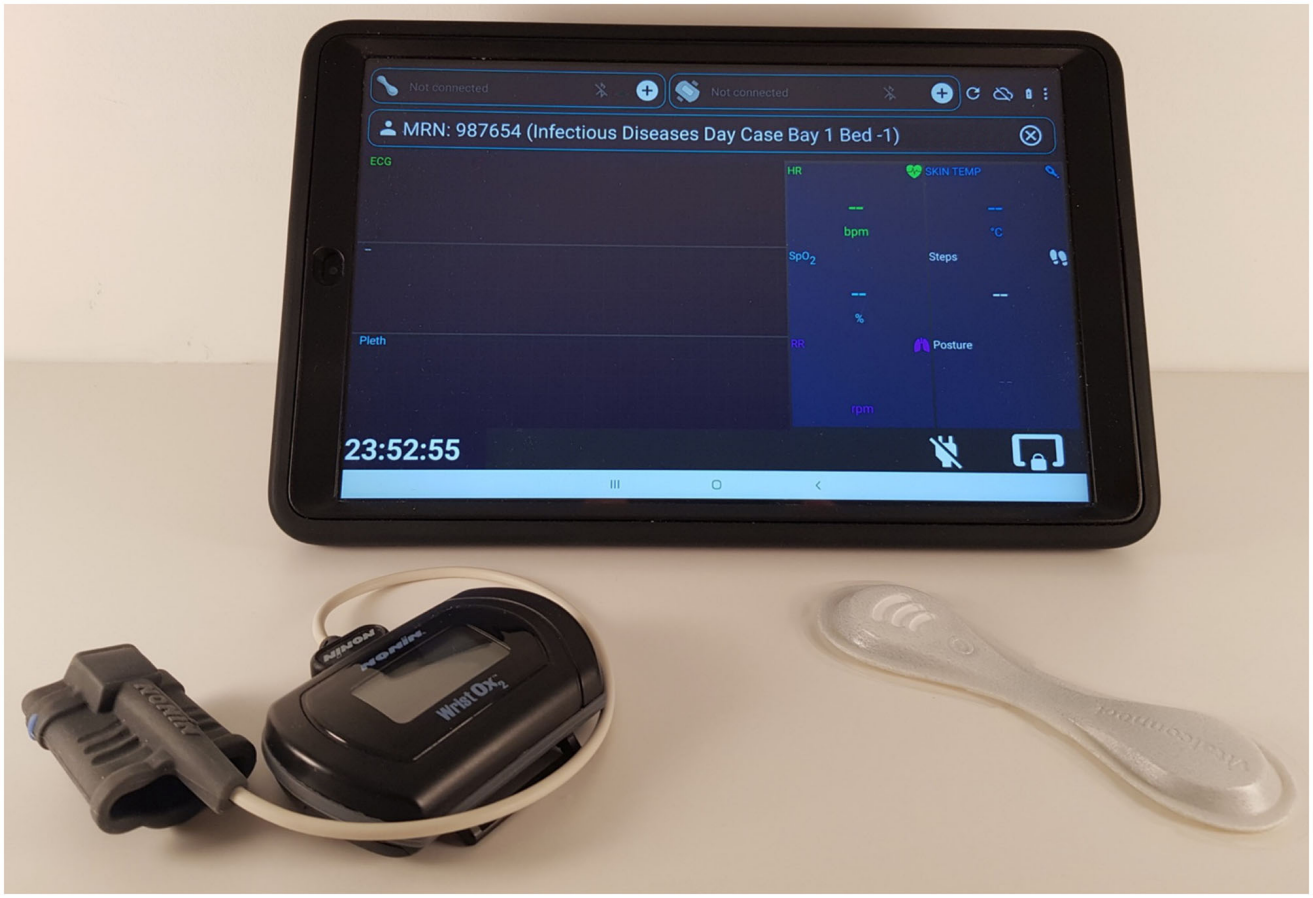
Hardware used to monitor COVID-19 patients on the isolation wards (from left to right): the Nonin finger-worn pulse oximeter, the Samsung Tab A 2019, 10-inch, with front and back protective casing, and the VitalPatch®.

While Nonin's BLE protocol documentation was provided by the vendor, so that it could be implemented by a third-party, VitalConnect only provided their Software Development Kit. The latter limits the VitalPatch® device to connect to only one Android Operating System (OS) tablet at a time (1-to-1 routing). As a result, BLE and Wi-Fi enabled Android tablets were the only choices for the patient data collection device required in our system use case. Wi-Fi technology allows the vital-sign data recorded with the wearable devices data to be transmitted from the Android tablets into remote servers *via* the hospital's Wi-Fi routers. The Samsung Tab A 2016 (SM-T585) and 2019 (SM-T515), 10-inch, models were selected for data collection (see Figure 1).

### Patient Data Collection App

**Figure 2:**
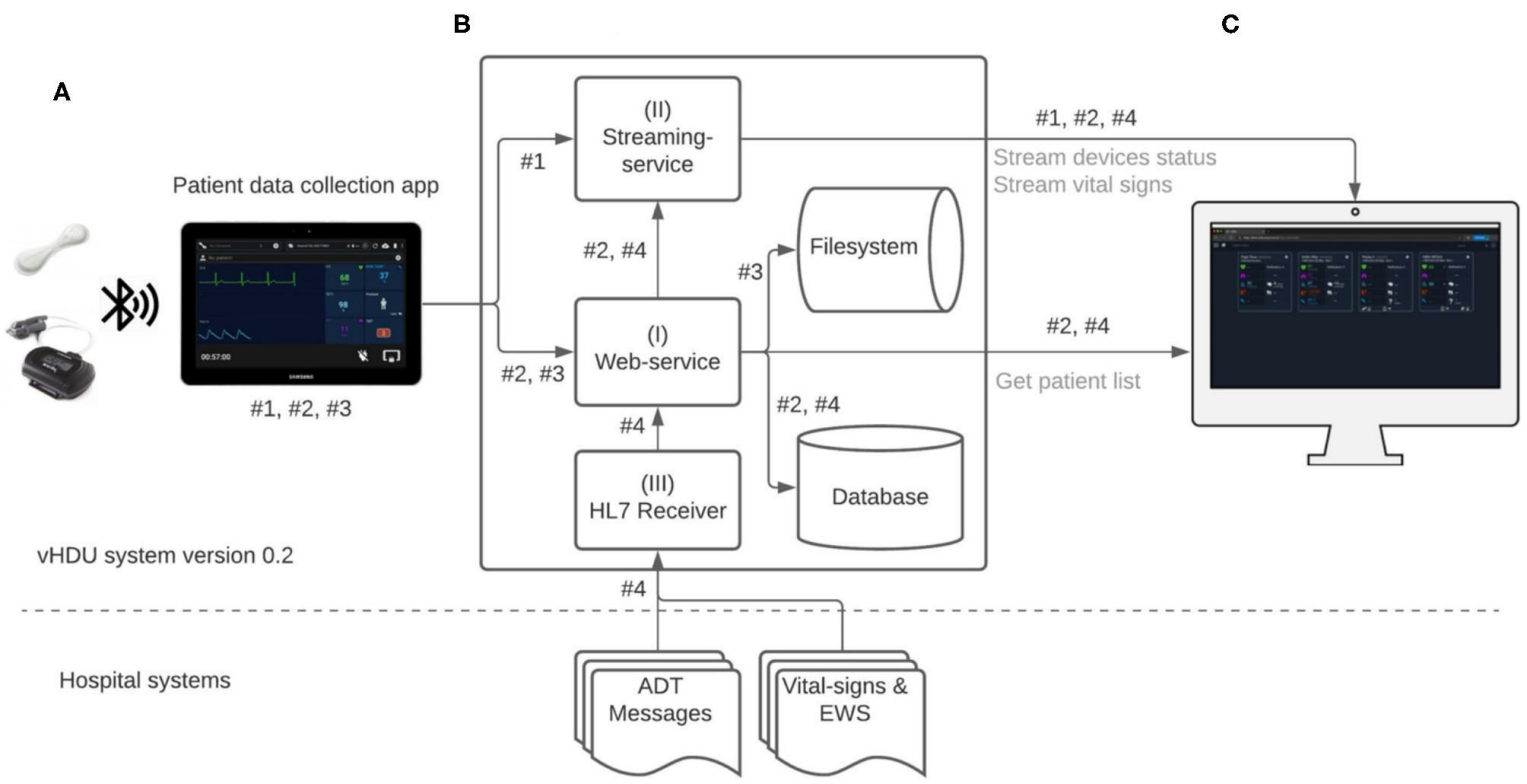
Virtual High-Dependency Unit (vHDU) system version 0.2 data-flow diagram. Components: **(A)** the Patient data collection tablet app, which retransmits the wearables data to the back-end; **(B)** the Web-application back-end, which stores and relays that information to **(C)** the Clinician Dashboard client, which displays the data from remote locations (*via* a web-browser). The main app datastreams of this system are: #1 - high-rate vital-sign values sent from the app to the streaming-service, each 2 s; #2 - high-rate 10-s median vital-sign values and the most recent wearable status values, sent each 10 s to the web-service, and low-rate 5-min vital-sign median values, sent each 5 min to the web-service; #3 - compressed files (ZIP format) with the wearables raw-data, sent to the web-service; #4 - HL7 (Health Level Seven standard) messages with e-obs chart data and patient Admission-Discharge-Transfers (ADT) information, which are sent from the hospital systems into a HL7 receiver application on the vHDU back-end. Data transmission from both the VitalPatch® and Nonin devices to the tablet app is encrypted using the Advanced Encryption Standard (AES) algorithm, over Bluetooth-Low-Energy. Data communication between the tablet app and the back-end web- and streaming-services, and between the latter and the Clinician Dashboard is encrypted using the Secure Sockets Layer (SSL) algorithm over the HTTPS and the secure Websockets protocols (for the web- and streaming-services, respectively). EWS, (local) Early Warning Score.

Figure 2 illustrates the data-flow diagram of the vHDU system. The Android Java app was the first component of the system to be designed. Our 1-patient to 1-tablet data routing approach presented a cost limitation, but also an opportunity: given that each tablet's computational resources are available for each patient, the first design decision was that as much of the patient wearable data pre-processing as possible would occur on the tablet app (rather than on the back-end server). Clinical staff from the COVID-19 wards were interested in reviewing remotely: (a) the patient vital-sign data and the connexion and battery status of the devices *in real-time*; and (b) the vital-sign times series trend *retrospectively*. Whilst (a) requires the processing of high-rate data, (b) requires that the data are summarised so as to make it feasible to both store it and allow the clinical staff to browse hourly to daily vital-sign trends (low-rate vital-sign data). The pre-processing strategies applied to the data collected in the app are described next.

#### Raw-Data Collection

To allow the detailed analysis and evaluation of the system retrospectively, all the numerical and waveform (raw) data from the devices were recorded in comma-separated values (csv) files on the tablet. Each 5 min, a batch of up to 10 files were compressed in ZIP format (a lossless data compression approach) and then sent to the back-end server (all files sent successfully to the server are then subsequently deleted from the app after 24 h). The raw csv files are recorded up to a maximum of 30 MB of data, corresponding to ZIP files of about 1.5 MB. We note that for each hour of patient monitoring the app creates about 7.5 MB of compressed files. The app's raw-data upload to the back-end is represented by datastream #3 in Figure 2. The corresponding web-service is described in section Web-Application Back-End.

#### High-Rate Data Collection

The synchronisation of the high-rate vital-sign data, and the connexion and battery status from the wearable and tablet devices, were implemented using the ReactiveX library (17). The latter uses a reactive programming paradigm, in which each sensor data (i.e., patch, pulse oximeter, and tablet data) is accumulated in its respective “Observable” variable, asynchronously, and for a given time window. For each variable and time window, either the last available data point or a summary statistic was determined (the median was used in our case, as it determines the most representative value in a given window, being less influenced by motion artefact than the mean). All the variables' simultaneous window values were then combined into a single (synchronised) data structure, using an appropriate “datastream operator” (see the “Zip” operator, from the ReactiveX library).

The following high-rate numerics datastreams (illustrated as datastream #1 and datastream #2 in Figure 2, respectively, and detailed in Table 1) were pre-processed on the tablet (as described) and then sent to the back-end:

i. for each 2-s window, the most recent available numerics (HR/PR, RR, SpO_2_, number of steps and patient posture);
ii. for each 10-s window, high-rate numerical estimates, i.e., median HR/PR, RR, and SpO_2_, the most recent number of steps and patient posture, and the wearable/tablet device status data (i.e., the available storage space, battery status, BLE and Wi-Fi connexion status), pre-processed into a (10-s) “data-packet”.

**Table 1:** Technical specification of the datastreams sent from the app or from the e-obs hospital system into the vHDU system version 0.2.

**Datastreams^**a**^**	**#1**	**#2**		**#3**	**#4**
Protocol	Socket.io	HTTPS	HTTPS	HTTPS	MLLP
Data source	Wearables	Wearables	Wearables	Wearables	e-obs
Data frequency	2 s	10 s	5 min	5 min	~ 4 h
Data format	JSON	JSON	JSON	ZIP	HL7 v2.6
Data window size^b^	2 s	10 s	5 min	variable (up to 30 MB)	
Synchronised	✓	✓	✓^c^		✓
On clinician dashboard	✓	✓^d^	✓		✓
Wearable Status					
Connexion		Most recent			
Battery		Most recent			
Vital Signs					
Heart rate	Most recent	Median	Median	Raw	✓
Respiratory rate	Most recent	Median	Median	Raw	✓
SpO_2_	Most recent	Median	Median	Raw	✓
Blood pressure					✓
Temperature					✓
Oxygen therapy					✓
AVPU/GCS					✓
Steps	Most recent	Most recent	Most recent	Raw	
Posture	Most recent	Most recent	Most recent	Raw	
Waveforms					
Electrocardiogram				Raw	
Photoplethysmography				Raw	
Acceleration (3-axis)				Raw	

*“most recent” - the latest data point available in that window is used. “median” - the median value is determined over a given window. “raw” - all the (raw) data from the wearables are collected*.

a *The datastreams numbers match those used and illustrated in Figure 2*.

b *The data windows do not overlap*.

c *The 5-min medians are computed from the 10-s median values, which are synchronised as detailed in section High-Rate Data Collection*.

d *The vital signs from the 10-s data-packet are only visible in the Clinician Dashboard in case the 2-s data are absent*.

*AVPU, Alert, Voice, Pain, Unresponsive scale; GCS, Glasgow Coma Scale; e-obs, hospital electronic observation system used by nurses to enter vital-sign observations; MLLP, Minimal Lower Layer Protocol; JSON, JavaScript Object Notation*.

The 2-s data-rate requirement came from feedback from staff on the Cardiology wards (where our vHDU system was also to be deployed), who need to track the vital signs of cardiac patients in (near) real time. At such a high rate, it is possible to replicate the real-time view from the Android tablet, located at the patient bedside, on remote interfaces. This was also felt to be an important feature for monitoring COVID-19 patients on the isolation wards as it was known that the virus could cause very rapid deterioration of a patient's cardio-respiratory system. Similarly, clinical research and ward staff confirmed that updating the device connexion and battery status every 10 s would be sufficient. We note that the high-rate vital-sign data are not filtered (e.g., for motion artefacts) as the objective was to display remotely real-time data from the devices.

#### Low-Rate Data Collection

To allow browsing large periods of vital-sign data from a remote client, the research team decided to limit the resolution of the low-rate data to 5-min windows. The low-rate data are observed retrospectively and may be significantly influenced by motion artefact. Therefore, to remove as many as possible of the motion artefacts and ensure that each data channel buffer would not consume too much memory, the vital-sign data was first summarised for each 10-s window and subsequently summarised further for each 5-min window, using the median estimator in both cases. Five-minute median values of HR/PR, RR, and SpO_2_, and the most recent number of steps and patient posture in those windows, were therefore estimated from the (non-overlapping) 10-s medians aggregated from datastream described in item (ii), section Raw-Data Collection, and finally sent to the back-end server.

The 10-s and 5-min datastreams were recorded both in the app and the back-end databases, generating ~200 KB of data per patient monitoring hour (both detailed in Table 1 and illustrated as datastream #2, in Figure 2, as the second is derived from the first on the app, and then sent and stored on the same back-end configuration, see also Table 2). This is negligible when compared with the amount of raw-data recorded by the app. Finally, the app database is cleared every time a patient is disconnected from the vHDU system by the clinical staff.

**Table 2:** Technical specification of the system deployed in the isolation ward.

**Software**	**Framework (language)**	**Server 1**	**Server 2**
Patient data collection app	Android v28 (Java v8)	-	-
Streaming-service^a^	ExpressJS; Socket.io (Javascript)	Node.js v12	
Web-service^b^	CakePHP v3 (PHP v7.3)	IIS v10	PostgreSQL v12
File upload web-service^c^	CakePHP v3 (PHP v7.3)	-	IIS v10; PostgreSQL v12
HL7 receiver	Apache Camel v2; Spring Framwork v4 (Java v8)	Apache Karaf v4	PostgreSQL v12
Clinician dashboard	ReactJS v16 (Javascript)	IIS v10	-

*Servers 1 and 2 are both virtual machines with 8 GB of RAM, 4 CPUs each and the main application containers running the vHDU system components are identified for each server*.

a *Streaming-service configuration to relay datastream #1, and the 10-s data-packets from datastream #2, to the Clinician Dashboard*.

b *Web-service configuration to receive and process datastream #2 in Figure 2, which includes both the 10-s and 5-min data-packets processed in Server 1 and stored in Server 2*.

c *Web-service configuration to receive and process datastream #3 in Figure 2, related with the raw-data files (ZIP format) that are uploaded and stored in Server 2 each 5 min*.

*IIS, Internet Information Services; HL7, Health Level Seven standard*.

#### App Interface

**Figure 3:**
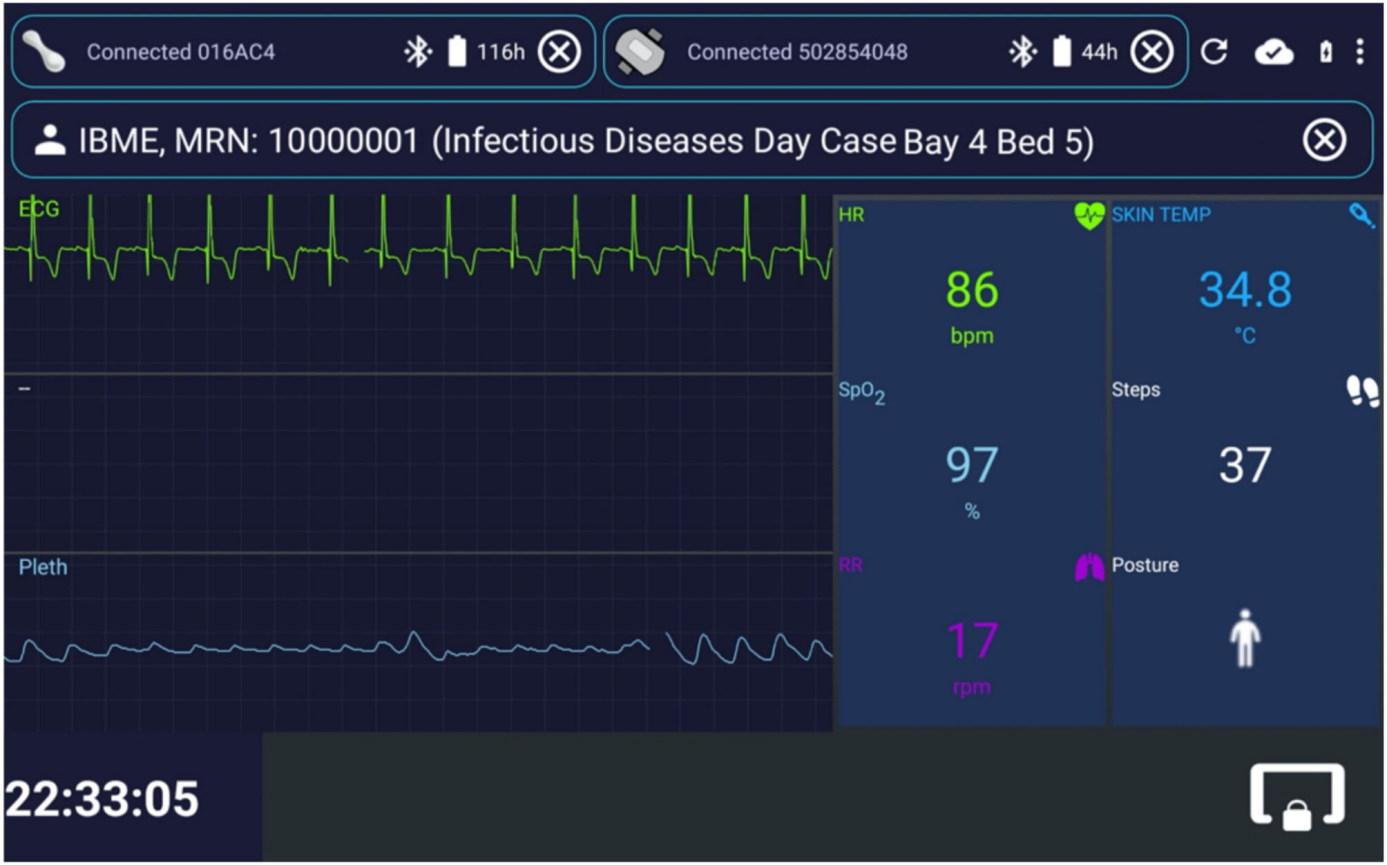
Interface for the patient data collection app, implemented in Android Java. Exemplar test data are shown. The status of the wearable device can be observed at the top of the image, with both the VitalPatch® and the Nonin devices in “Connected” state, and with serial numbers “016AC4” and “502854048,” respectively. The top right icons enable (from left to right): restarting the app; checking the connexion status with the back-end server; checking the charging and battery status; and entering the app settings. The patient can be unregistered by pressing the outlined “ × ” icon on the right of the patient information field. The live vital-sign and waveform data (ECG, Electrocardiogram, and Pleth, Photoplethismography) values are displayed on the right side and left side of the app, respectively. The lower right icon locks the app screen, leaving only the lower left clock displayed. In this case, the monitoring and streaming of the vital-sign data occur in the background, likely to be a less distracting mode for patients (particularly useful at night, because of the light emitted by the tablet). IBME, Institute of Biomedical Engineering; MRN, Medical Record Number; bpm, beats per minute; rpm, respirations per minute; HR, Heart Rate; RR, Respiratory Rate; TEMP, Temperature; SpO_2_, peripheral blood Oxygen Saturation.

Figure 3 shows exemplar test data displayed in the patient data collection app interface. It was designed to mimic the functionality of bedside patient monitors. Once the tablet registers with the web-application back-end, clinical staff can enrol a patient in the AMS *via* their wristband Medical Record Number (MRN) and location (i.e., ward, bay and bed, when the last two are available). A monitoring session is then created on the back-end, and data from one VitalPatch® and one Nonin devices can be linked and collected *via* the app. From top to bottom in Figure 3: the VitalPatch® and Nonin connexion status and battery (in hours) are displayed on the top left and right corners, respectively; the patient MRN and location are displayed next; the waveform-grid (ECG in green and PPG in blue) and the numerics-grid (HR, RR, SpO_2_, skin temperature—removed when deployed at the isolation wards as it was not felt by the clinical staff to be a reliable indicator -, number of steps and patient posture) can be observed in the middle left and right of the display, respectively.

### Web-Application Back-End

The vHDU system back-end (Figure 2B) was developed to receive and store the patient data collected by the app and make them available to client interfaces on remote locations. It consists of three main services (referenced in Figure 2B, using the same numerals):

- The web-service, which uses the CakePHP framework Model-View-Control (MVC) software architecture and the Representational State Transfer (REST) approach for its Application Programming Interfaces (APIs). This web-service receives the 10-s and the 5-min app data, both illustrated as datastream #2, in Figure 2, as both are further processed on the back-end and stored on a relational database (PostgreSQL). While the first is relayed to the clinician dashboard *via* the streaming-service, the second is made available to the same client interface *via* the REST API (e.g., when a particular patient chart is reviewed). The raw-data compressed files are also received by the web-service, each 5 min, and stored on the back-end filesystem (illustrated by the datastream #3, in Figure 2). Secure access to the web-services resources is maintained *via* the use of Java Web Tokens (JWT).
- The streaming-service: the Node.js framework was used to develop the real-time communication of the 2-s high-rate numerical data from the app to the remote interfaces. This real-time communication is illustrated as datastream #1 in Figure 2, being present in the arrow from the app component to the streaming-service, and then in the arrow from the latter to the Clinician Dashboard, in that figure. Note that the 2-s data are not stored on the back-end. Additionally, the 10-s data stored in back-end, are also relayed from the web-service (I) to the remote interfaces *via* the streaming-service. This real-time communication is also represented in Figure 2, first in the arrow from the web-service to the streaming-service, and then in the arrow from the latter to the Clinician Dashboard, in that figure. The socket.io protocol is used for the real-time, bi-directional communication. JWT is also used to authenticate the initial socket.io handshake request.
- The HL7 receiver: the development of this component was based on prior work from the research group in consuming HL7 (Health Level Seven standard) messages from the hospital systems (18). Apache Java Camel is used to receive (*via* Minimal Lower Layer Protocol, MLLP), store and process the HL7 messages from the hospital's Admission-Discharge-Transfers (ADT) system and from the hospital system responsible for the electronic notification and documentation of vital-sign observations (e-obs) by clinical staff (19), also known locally as the electronic Track-and-Trigger (e-T&T) chart. Finally, a Java Spring REST API makes these data available to the web-service back-end, so they can be stored alongside the continuous wearable data transmitted by the tablet app. The data-flow of this component is illustrated as datastream #4 in Figure 2.

### Clinician Dashboard

The Clinician Dashboard (Figure 2C) was designed to allow the review of both the real-time vital-sign and device status data, from multiple patients in parallel, and the 5-min vital-sign trend charts, for each patient, on the remote ward. The ReactJS (JavaScript) framework was used to develop an interface that could display both the high- and low-rate datastreams *via* a web-browser (usually available from any hospital computer), which includes:

- The staff accounts (secured *via* username and password credentials) and patient details administration pages. Staff accounts are pre-configured with a set of hospital wards from which they are able to access data.
- A homepage, which allows authorised clinical staff accounts to browse a list of patient cards from their pre-configured set of hospital wards. The web-service APIs allow this list to be queried, whilst the socket.io protocol allows the streaming of the device status data every 10 s, and of the real-time vital-sign values, every 2 s, to each patient card. For each patient card, the most recent values from the continuous datastream (HR, RR, SpO2, number of steps, and patient posture) are displayed alongside the most recent complementary nurse observations, collected *via* the HL7 receiver (BP, Temp and level of consciousness). Temp, the core body temperature, is recorded by the nurses during their observations using a tympanic thermometer, when they also assess the patient's level of consciousness using the Alert, Verbal, Pain, Unresponsive (AVPU) Scale, or the Glasgow Coma Scale (GCS).
- An *augmented e-obs* chart, with the full set of vital-sign data for each patient, combining the (typically) 4-hourly vital-sign observations from the nurse, and corresponding (local) Early Warning Score, with the 5-min median vital-sign values (HR, RR, SpO_2_) from the wearable AMS (the wearable data are not scored at this point).

**Figure 4:**
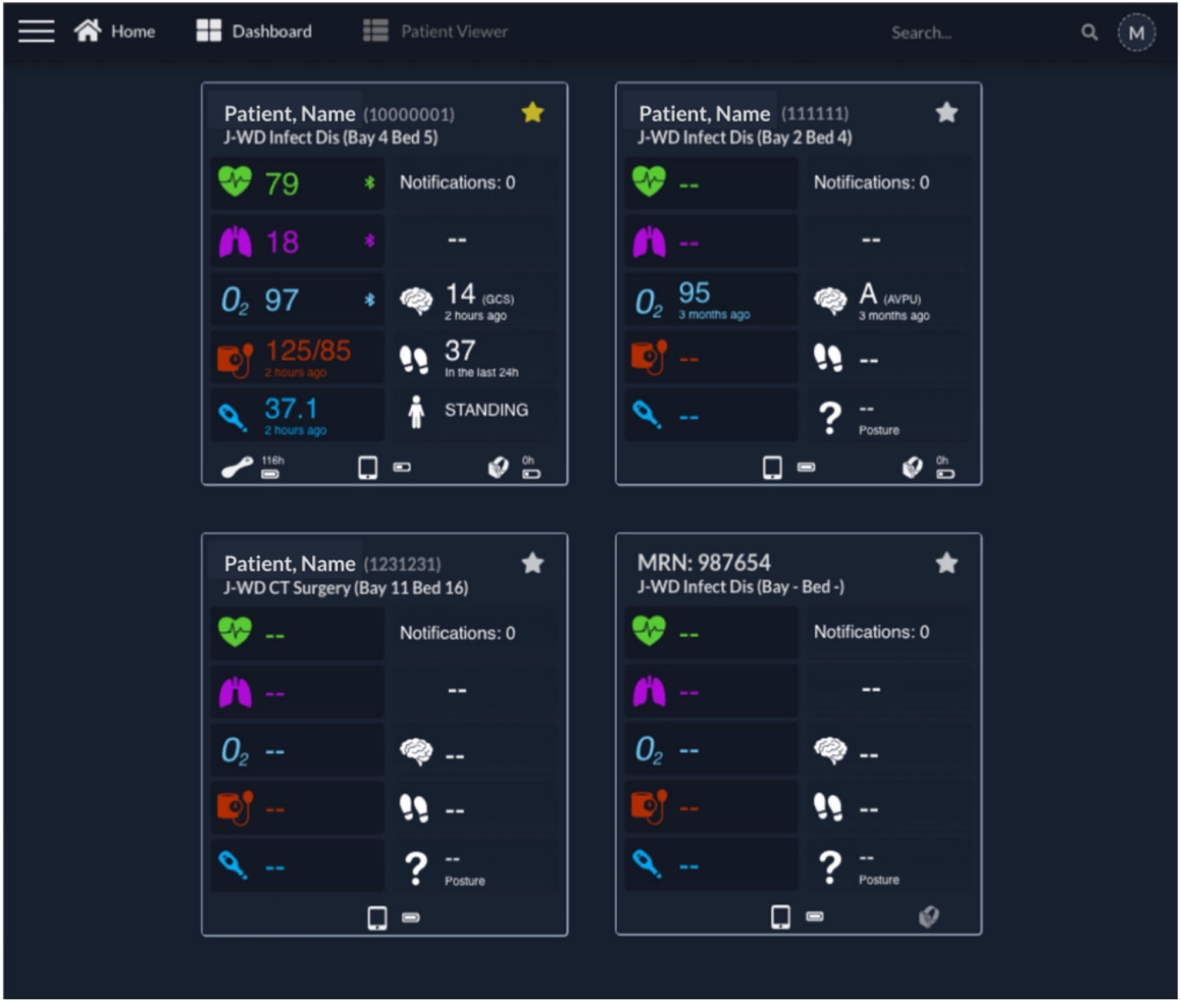
Clinician Dashboard homepage with four exemplar test patient cards. Each card presents on the left: the Heart Rate, Respiratory Rate, peripheral blood Oxygen Saturation (“O_2_”), Blood Pressure, and Temperature; and on the right: the Notifications (disabled at this time), continuous Early Warning Score (disabled at this time), Level of consciousness, Steps and Posture. The patient name, Medical Record Number and location are shown at the top of each card. At the bottom of each card, the status of the wearables, including connectivity and battery status, can be observed (from left to right, the VitalPatch®, tablet, and Nonin icons are shown when present—such as in the first card). It can be noted that the upper left patient is “Starred” (by the active yellow star), with a VitalPatch® device connected, in range and with a remaining battery of 118 h; the tablet has a battery charged at 50% of capacity; and the Nonin device is connected to the tablet but has no battery left (0 h). The battery icon flashes in this case, to prompt for a change of batteries. The upper right patient had their last observation 3 months ago, showcasing the system's ability to keep the last patient state until they are discharged from the system (the Nonin also has no battery left in this case). The lower left patient has been pre-registered but the wearable devices have not yet been attached to the patient, as indicated by the lack of icons for the wearables, and thus no vital-sign data are available yet. The lower right patient has no “Patient, Name,” being identified only by the MRN in this case. The right-side Nonin icon is also greyed, representative of a Nonin pulse oximeter associated with the patient but out of range from the patient's assigned vHDU tablet. AVPU, Alert, Voice, Pain, Unresponsive scale; GCS, Glasgow Coma Scale; J-WD, John Radcliffe Hospital Ward Code; MRN, Medical Record Number.

Figure 4 shows the implemented Clinician Dashboard homepage, which allows authorised staff accounts to review the active patient monitoring sessions from a computer screen located at the nurse bay (the combination of the vHDU system components described up to this point fulfils the third requirement from section Introduction). Each card corresponds to one patient monitoring session with data transmitted by the patient data collection app. The patient is identified at the top by their first/last names, MRN number and location (ward short code/bay/bed). Real-time wearable data are displayed alongside the complementary intermittent vital-sign observation data.

The vital-sign timing is shown below its value in case the values are older than a minute. The wireless connectivity of the wearable devices and their battery status are shown at the bottom of each patient card. Finally, the patient card list can be filtered by searching for their details, from the search box, at the top right corner of the page header, or filtering by starred patients (note the yellow/white stars at the top right corner of each card).

**Figure 5:**
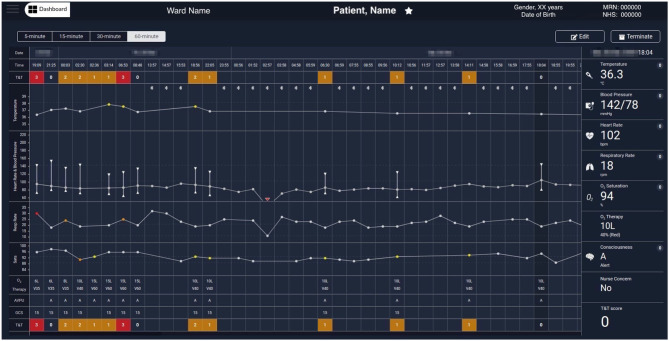
Exemplar augmented e-obs chart from the Clinician Dashboard, showing wearable and e-obs vital-sign data from a COVID-19 patient in the isolation room. The header provides the patient's demographics (from left to right): the ward, bay and bed location, the name, gender, age (with date of birth below), and the MRN and NHS codes. The clinicians have the option to “Edit” the patient information, or “Terminate” the patient's session in the vHDU system (automatically unregistering them from the assigned tablet). The clinicians can review the periodic wearable data, i.e., the 5-min median estimates, using 15-, 30-, or 60-min windows (the most recent 5-min estimates in each window are shown), alongside the intermittent e-obs data (usually entered 4-hourly by the nurses), to facilitate tracking of the patient state. Wearable data is distinguished from the e-obs by using the Bluetooth sign at the top of the Temperature row. Only the e-obs data have scores associated with it, presented in the T&T row at the top and bottom of the chart. In this hospital setting, an Early Warning Score greater or equal to 3 was coloured red, denoting high risk. In this exemplar patient chart, only nurse observations (e-obs data) are present at the start, showing low SpO_2_ (89% at 2:30 AM) and high RR, high Temp, and the use of oxygen therapy (26 rpm, 37.9°C and Venturi mask 60, V60, at 15 litres per minute, respectively, at 6:35 AM). The wearable data is summarised in hourly windows to allow the review of a longer monitoring period. It is possible to observe that during this period the VitalPatch® and the Nonin devices were fitted to the patient from 13:00 and then temporarily removed between 16:00 and 23:00. Periodic VitalPatch® data was then consistently available for remote monitoring of the patient status from the nurse bays; however, the Nonin was still removed often by the patient during that period (staff continued to try to use it to monitor the patient's continuous oxygen therapy, which can be confirmed by the presence of the Venturi mask, V40, in the 4-hourly e-obs data present during this period). MRN, Medical Record Number; NHS, National Health Service; T&T, Track & Trigger; Sats, peripheral blood Oxygen Saturation; AVPU, Alert, Voice, Pain, Unresponsive scale; GCS, Glasgow Coma Scale; RA, Room Air; O_2_, Oxygen.

Figure 5 shows the augmented e-obs chart for an exemplar COVID-19 patient, presenting the vital-sign observation sets recorded by the nursing staff alongside the 5-min vital-sign data estimates acquired using the wearables (meeting the fourth requirement from section Introduction). These two different data sources can be identified on the chart by the absence or presence of the BLE symbol at the top of the temperature row, respectively. Note that the column of the nurse-recorded observation set, at 18:04, is selected and the respective Temp, BP, HR, RR, SpO_2_, oxygen therapy, AVPU, and (local) EWS values recorded at that time, are shown in the rightmost column.

**Figure 6:**
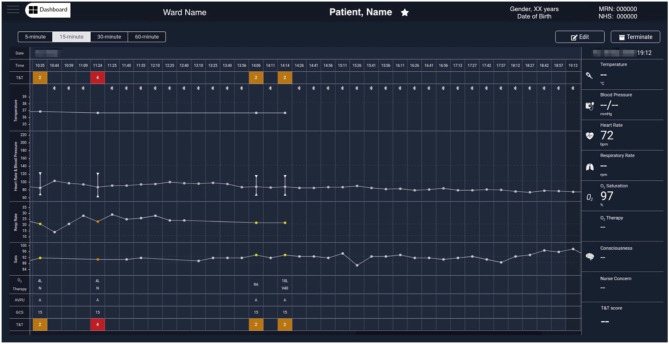
Exemplar augmented e-obs chart from the Clinician Dashboard, showing wearable and e-obs vital-sign data from a COVID-19 patient in the isolation room. In this example, the wearable data from 10:44 to 19:12 is summarised in 15-min windows (i.e., the “15-min” view was activated, and only the most recent available 5-min median estimates within each window are displayed), and shown alongside the intermittent e-obs data. Only the e-obs data had scores associated with it, presented in the T&T row at the top and bottom of the chart. In this hospital setting an Early Warning Score (EWS) greater or equal to 3 was coloured red, denoting high risk. In the period displayed in the chart, the VitalPatch® was already fitted. The increase in the VitalPatch® RR, might have triggered the nurse review of the patient 1 h after the previous review (usually done 4-hourly when the previous total EWS is 2), as an additional e-obs set was entered by the nurse at this time, with high RR (24 rpm), low SpO_2_ (90%) values and a total EWS of 4 (1 h after the previous e-obs set, at 10:35 AM). We note that at this point the patient was receiving oxygen therapy *via* a nasal cannula (N) at 4 litres per minute. It is possible to observe that, in the subsequent 3 h, the VitalPatch® was removed and the Nonin pulse oximeter fitted to the patient to monitor the SpO_2_ while the oxygen therapy was escalated, by applying a Venturi Mask 40 (V40) at 10 litres per minute, at 14:14. A recovery trend can be observed, with HR and SpO_2_ estimates from the Nonin pulse oximeter stabilising in the evening at 72 bpm and 97%, respectively (displayed at the end of the chart). Note that a nurse e-obs set is absent during this period in the chart (i.e., between 14:14 and 21:23, the time at which the patient was removed from the vHDU system; the chart only shows up to 19:12). MRN, Medical Record Number; NHS, National Health Service; T&T, Track & Trigger; Sats, peripheral blood Oxygen Saturation; AVPU, Alert, Voice, Pain, Unresponsive scale; GCS, Glasgow Coma Scale; RA, Room Air; O_2_, Oxygen.

Figure 6 shows another COVID-19 patient data example in which a 5-min median values, determined from the wearable data, are selected, at 19:12 (a data-packet with only HR and SpO_2_ from the Nonin, in this case). Displaying the wearable data interleaved with the nurse observations facilitates the review of the patient physiology trend by staff. Wearable data estimates can be collapsed to show only the (most recent 5-min) estimates at 15-min, 30-min or hourly intervals, to make it possible to review longer time-series more easily, when needed.

### Fault-Tolerant Software Strategies for Continuous Monitoring

To optimise the wearable AMS prior to patient use, several rounds of testing and troubleshooting were performed in 2019, with the ward staff wearing the devices during working hours. This allowed for software cheques, connectivity assessment, and integration of the wearable AMS into the clinical environment. The testing was also intended to encourage AMS familiarisation amongst ward staff.

Reliability of the AMS was an important feature, as it is critical that the nurses do not spend time investigating problems caused by loss of sensor data, as described in the fifth requirement from section Introduction. The following fault-tolerant software functionalities were implemented in the app to ensure its reliability for continuous monitoring of the patients' vital signs:

- a single app lockdown mechanism built into the Android OS, the “kiosk mode,” which “pins” the vHDU app to the foreground and limits the access to other tablet functionalities; this mode allows the Android OS to re-open the app automatically every time it closes;
- a background service that reconnects automatically to the BLE wearables, whenever intermittent connexion losses occur, up to 10 min, which can happen e.g., if the patient is very active or temporarily moves away from the tablet;
- a background service that forces the app to restart and reconnect if any connected wearable device is missing data for more than 10 min; this action resets the BLE connexion state at the OS level, and the app resumes the previously persisted patient- and wearable-registration state automatically.

These strategies guarantee that data loss is never >10 min from when an unaccounted software issue occurs in the app. Finally, the Clinician Dashboard was configured to reload automatically each 30 min (i.e., when loaded on a web-browser e.g., from the nurse bay). This ensures that its socket.io client regains/maintains connectivity with the streaming-service, and consequently keeping the patient cards data updated.

## Deployment of the AMS on the Isolation Ward

In the previous section we detailed the Oxford vHDU AMS functionalities supporting the first five requirements deemed essential to allow the remote monitoring of the vital signs of COVID-19 patients in their isolation rooms, from the remote nurse bays. Next, we describe its deployment in the hospital's isolation ward. Our initial adaptation of the AMS for monitoring COVID-19 patients on this ward was completed in just 3 weeks, during March 2020. This isolation ward has a maximum capacity of 19 isolation rooms, with two nursing bays outside the isolation rooms. Four to five nursing staff and three to four medical doctors oversee the ward, in each of its two shifts (staff numbers being adjusted as required, in particular during the week-ends).

### Software Configuration

The patient data collection app was installed on 20 Android tablets, configured in “kiosk” mode and with Wi-Fi Protected Access 2 (WPA2) accounts. The web-applications back-end was deployed on one virtual server (except the compressed-files upload web-service), and an additional virtual server supported the databases and the compressed-files storage. Their technical specifications can be reviewed in Table 2. Both tablets and servers were behind the Oxford University Hospitals (OUH) National Health Service (NHS) Foundation Trust firewall. The transmission of HL7 v2.6 messages over MLLP is not encrypted. Therefore, an additional requirement to having our servers only available from the hospital intranet, was that they ran a bespoke anti-virus software and were managed by the hospital cyber-security team. All data communication between the tablet app and the servers was encrypted using the Secure Socket Layer (SSL). The VitalPatch® and Nonin data communication was also encrypted using the Advanced Encryption Standard (AES) over BLE. Finally, the Clinician Dashboard accounts were pre-configured to show data from the isolation ward only. These configurations helped in achieving the essential data security and patient confidentiality standards of the sixth requirement from section Introduction. Finally, we note that patient data collected from an electronic system that is part of a research study, such as the vHDU system, is required to be deleted from the hospital servers within a period of 5 years from the completion of the study.

### Hardware Configuration

The AMS hardware (tablets and wearables) was configured to minimise the risk of the vHDU equipment spreading infection and to facilitate its setup in the patients rooms:

i. the VitalPatch® is a single-use device, and safely disposed of after usage;
ii. one Nonin pulse oximeter is paired exclusively with one Android tablet, and its serial number tagged on the tablet. The serial number is broadcast to the tablet app, *via* BLE, allowing staff to easily connect both. After use, the casing and finger-probe of the device are cleaned with appropriate wipes and the wrist-straps are replaced with newer ones, for the next patient (the old straps were disposed in this case);
iii. tablets are covered with a back and front protective casing; their app pre-configured with the ward, bay and bed information; while in use, tablets are deployed near a power-socket and within the reach of the Wi-Fi signal in each isolation room; after use, they are cleaned with appropriate wipes (or the casing replaced, as required) and, when not in use, they are secured in bags at the nurse bays;
iv. a dedicated computer screen is configured in the main nurse bay to display the Clinician Dashboard, which allows authorised staff to review the vital signs of multiple patients, as well as the status of their wearable devices and tablet. The dashboard is a web-application front-end, available from any ward computer screen with a modern web-browser within the hospital intranet.

### Training and Maintenance

The vHDU system optimised for vital-sign monitoring of patients on the isolation ward was deployed on 20th March 2020 and the first patient was registered in the system on 23rd March (20). Two one-hour training sessions per week were given by a research nurse (LY) and a research engineer (CR), for the 1st month following the start of the first wave of the pandemic, to cover the entire staff. Between March and August 2020, the biomedical research engineers (MDS, CR and MAP) and one software engineer (RL), worked continuously to provide equipment, system configuration and software improvements. The clinical research staff (SV, CA, and LY) received weekly feedback from the nurses managing the COVID-19 patients.

The ability to calibrate the VitalPatch® RR estimates manually, *via* the tablet App, was added 2 weeks after the initial deployment. This device needs to identify the patient posture—standing vs. lying down—to select the correct accelerometer axis and start deriving RR (21). Prior to that, the patient had to take about 30 steps for the RR estimation to be calibrated, which did not happen if the device was fitted while the patient was in bed, and remained in bed.

Finally, the integration of the 4-hourly nurse observations vital-sign values into the Clinical Dashboard was completed and deployed from the middle of June 2020 (i.e., only continuous data was shown in the patient cards and augmented chart up to this point), allowing staff to review both sources of vital-sign data in one interface. This led to the system architecture in Figure 2: vHDU version 0.2.

## Results

### System Uptake on the COVID-19 Isolation Ward

Preliminary feedback regarding the uptake of the vHDU AMS on the isolation wards was received mainly from clinical staff during the training and system maintenance sessions offered by the research team. It was not possible to interact with/interview the patients during the pandemic. Feedback on the AMS usage during the first wave can be categorised as follows:

- VitalPatch®–The disposable cardiac patch was well-received by both the clinical staff and the patients. Staff found it easy to fit on the patient and connect to the tablet App, and that it would not fall easily from the patient's chest. Clinical research staff observed that HR and RR signal quality deteriorated (i.e., noticeable by intermittent HR and absence of RR estimation) when the patch was applied on patients from whom it was not possible to clear the chest hair completely.
- Nonin—Clinical staff reported that some patients found it difficult to comply with using this device continuously. This confirmed our wearability study findings (11), in which finger-based pulse oximeters such as the Nonin, were found to be less comfortable and tolerated than the cardiac patches, and ring-shaped or wrist-only pulse-oximeter devices. However, the Nonin was the only one capable of regaining connectivity without nurse intervention and with confirmed clinical-grade accuracy. Clinical staff also reported to have compared the Nonin SpO_2_ estimates with those from a Dinamap vital-sign monitor used in the ward, in the first couple of weeks. Our hypoxia study (13) showed that although the Nonin was within the clinical-grade accuracy guidelines (i.e., Root-mean-squared error ≤ 4%), it showed a negative bias of −1.92 (±2.73)%, and motion significantly deteriorates its estimates. The device was ultimately compulsory to use for those COVID-19 patients requiring oxygen therapy (usually less active and lying in the room beds), and therefore requiring remote monitoring of their oxygen levels.
- Linkage with the e-obs system data—displaying the 4-hourly nurse observations alongside the wearables estimates in the Clinician Dashboard was well-received by staff. However, the manual introduction of the MRN (from the tablet App or from the Dashboard) was found to be prone to error, as in a couple of instances the incorrect number was introduced, which prohibited the capture of the intermittent vital-sign data from the HL7 receiver system. Vital-sign data capture systems usually use a barcode scanner to acquire the patient MRN (or other hospital identifier) automatically from the patient wrist-band (avoiding error). It was not possible to have such a solution ready for the AMS, as the patient enrolment was often made *via* the tablet app and it would have potentially required a barcode scanner per isolation room. To avoid this error, future approaches will use a combination of visual cues and a camera-based barcode scanner approach (*via* camera-enabled tablets).

Figures 5, 6 show wearable and e-obs data collected by the vHDU system from two different COVID-19 inpatients in isolation rooms. The wearable data are shown for hourly- and 15-min windows, respectively. Both patients received oxygen therapy to compensate for the low SpO_2_ (<90%), and high RR (>20 rpm). The first patient maintained a low SpO_2_ throughout the displayed period, requiring additional 4-hourly nurse observations to adjust therapy. In contrast, the second patient showed two relevant events in the wearable data: (a) an increase in the VitalPatch® RR estimate (displayed at 10:09), which might have triggered a nurse review at 11:24 (resulting in a total EWS of 4 at that point, indicating deteriorating physiology), 1 h after the previous nurse review; (b) a recovery trend towards the end of the period displayed on the chart, the Nonin pulse oximeter reporting SpO_2_ and HR values of 97% and 72 bpm, respectively. For the latter patient, the nurse did not perform additional observations during the afternoon period (between 14:14 and 21:23, the time at which the patient was removed from the vHDU system—the figure only shows up to 19:12), indicating that, as long as the patient showed a recovery trend *via* the wearable system, the nurse could make the decision not to enter (with full PPE) the isolation room.

The system has now also been used during the second and third waves of the pandemic in the UK (November 2020—April 2021). Since April 2021, 20 semi-structured interviews regarding its use have been held with members of the nursing staff with experience of the system. These interviews were held *via* telephone or face-to-face, and used purposive sampling to gain as wide a range of views as possible. The findings from these interviews will be reported in a subsequent paper.

### Continuous Data Available for Remote Monitoring on the COVID-19 Isolation Ward

Preliminary results regarding the continuous data captured by the AMS, and available for the remote monitoring of COVID-19 patients on the isolation rooms, during the first wave of the pandemic in the UK, was reviewed as part of the quality improvement project (Audit Datix ID Number 5973), approved by the OUH Trust on 8th April 2020. These results were assessed by calculating the total amount of data acquired by each device, the median amount of data collected per patient and the number of active monitoring sessions per day. The latter has been compared with the number of new COVID-19 cases per day in England (22) and the number of COVID-19 related hospital admissions in England (23).

A total of 59 patients were monitored *via* wearable devices between 20th March 2020 and 2nd August 2020. The system monitored patients for a total of 2,938.8 h, corresponding to 88.1 [62.5, 94.5]% of the median time the patients were registered in the system (Table 3). This corresponds to a median of 31.5 [8.8, 75.4] hours of vital-sign data per patient (minimum was 20 min and maximum was 10 days). The VitalPatch® HR contributed the most data with 1,930 h. The amount of RR data was about 16% lower, mainly because of the 2-week delay in the introduction of the manual re-calibration step required to start its estimation from nurse input. The amount of SpO_2_ data is about 21% lower than that of the HR because some patients found it difficult to be compliant with the wearing of the finger-worn Nonin probe. Staff would only enforce the wearing of the probe if the patient was given oxygen therapy. When the VitalPatch® data was not available, the Nonin PR estimates would provide the HR measurements on the dashboard.

**Table 3:** Statistics on the vHDU system usage in the John Radcliffe Hospital isolation ward, between the 20th of March and the 2nd of August 2020.

**Metric**	**Period: 20/03 – 02/08/2020**
# Patients monitored	59
# Monitored hours	2,938.8
Median monitoring hours [Q1, Q3]	31.5 [8.8, 75.4]
Median monitoring hours [Q1, Q3] (%)	88.1 [62.5, 94.5]
# Nonin SpO_2_/Pulse Rate hours	1,500.5
# VitalPatch® Heart Rate hours	1,930.0
# VitalPatch® Respiratory Rate hours	1,649.9
Max number of patients simultaneously monitored	9

*Q, Quartile*.

**Figure 7:**
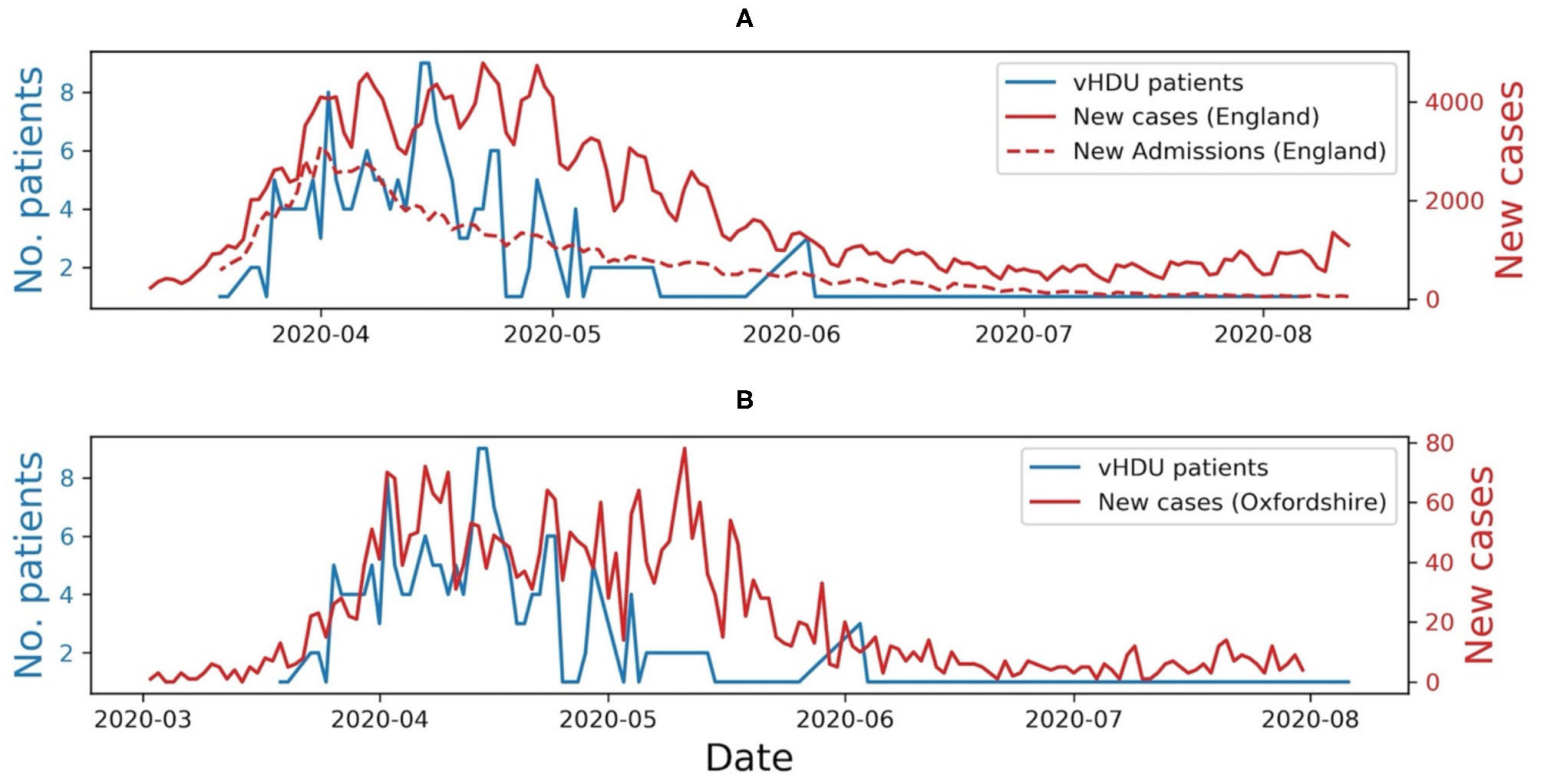
**(A)** Number of COVID-19 patients monitored *via* the virtual High-Dependency Unit (vHDU) system in the John Radcliffe Hospital isolation ward vs. the number of new daily COVID-19 cases and new daily COVID-19 hospital admissions, in England, between the 20th of March and the 2nd of August 2020. **(B)** The number of new daily COVID-19 cases in Oxfordshire, is shown for comparison. It can be observed that the system usage followed the trend of the first wave of the pandemic in the UK.

From observation of Figure 7, we can infer that at the peak of the first wave of the pandemic in the Oxford region, staff placed half of the ward patients on continuous monitoring (i.e., nine out of a total of 19 available rooms), using our wearable vHDU system.

Work is underway to analyse retrospectively the continuous vital-sign data collected during the COVID-19 pandemic, including the second and third waves (between March 2020 and April 2021) and corresponding patient adverse events (e.g., cardiac arrest calls, escalation of care, and mortality). This analysis will include (a) the frequency of nurse observations before and during the use of the vHDU system; (b) the relationship between instances of oxygen desaturation and associated respiratory and heart rate patterns; and (c) the relationship between the patterns of the vital-sign collected by the system and patients adverse events. We will also be reporting on the results of these analyses in a subsequent paper.

## Discussion

### Comparison With Other AMS

The challenges created by the COVID-19 pandemic to health services throughout the developed world has led to the accelerated deployment and acceptance of many remote monitoring technologies in the clinical setting (2). The work presented in this paper describes the adaptation of a wearables based AMS for real-time remote monitoring of the vital signs of COVID-19 patients being cared for on an isolation ward. The major difference between our prototype system and those reviewed earlier, is the assumption that nurse observations will be recorded using third-party interoperable software. These data are sent to the hospital Electronic Patient Record and made available to our system *via* HL7. Our system then displays it alongside the wearable continuous data.

We note that while the ViSi Mobile System only displays wearable device data, the Vista Solution® and Sensium®'s “E-Obs” package, allow staff to enter additional patient data in order to compute the EWS, generate alerts and suggest a clinical action. Our approach allowed clinical staff to retain their current e-obs system, but to review those data alongside the wearables data in one single chart (as illustrated in Figures 5, 6).

The 5-day battery life of the VitalPatch® adopted in our system, also helps minimise contact with patients in isolation rooms, when compared with the ViSi Mobile System modules which require daily charging. Finally, the Sensium® System does not include the continuous measurement of SpO_2_, the key parameter for managing COVID-19 patients, many of whom require oxygen therapy.

### Usage of the Oxford vHDU AMS During the First Wave

The need to undertake patient care on isolation wards while keeping clinical staff safe, and the focus of the local hospital teams (including clinical, engineering, and information technology) on helping the UK NHS cope with the COVID-19 pandemic created an environment which enabled the rapid adoption of our vHDU system into clinical practise. A number of factors helped in deploying the system in such a short time:

- a The prior selection of wearable devices that avoid the need for constant adjustment by nursing staff: amongst all the wearable pulse oximeters which we had previously investigated, the Nonin device was the only one that demonstrated good clinical accuracy, allowed consistent BLE communication and activated automatically once the patient's finger was positioned within the probe. Similarly, the results obtained with the VitalPatch® confirmed the conclusions from our previous wearability and functionality tests (11), as the patch was rapidly adopted on the isolation ward, with the advantage that it is a disposable device, thereby avoiding the risk of spreading the infection.
- b The use of interfaces familiar to OUH clinical staff: with minimal training, nursing staff when making their observations were able to review the outputs of the data collection app on the Android tablet as if it were a “bedside monitor;” additionally, the charts on the remote Clinician Dashboard were modelled on those used in the electronic observation system, widely used throughout the hospital.
- c The inclusion of fault-tolerant software mechanisms, e.g., to automatically recover from wireless disconnection of the wearable sensors and unaccounted software issues (with minimal data loss), to avoid nursing staff entering the isolation ward in order to make adjustments.
- d The close collaboration of the biomedical engineering and the clinical research teams with the ward staff (and vice-versa), enabled rapid feedback on the usage of the system, so that it could continually be iterated. During this period, biomedical expertise was required to improve the patient data collection app, e.g., in facilitating the setup of the VitalPatch® RR estimation, and to design and develop the patient chart displaying both the continuous and intermittent vital-sign data. The clinical research team validated the AMS functionalities with the ward staff.

The usage of the AMS on the isolation ward followed the trend of the first wave of the pandemic in the UK. Usage decreased when the number of COVID-19 related hospital admissions decreased (as shown in Figure 7A) and when other hospital wards were reorganised to make them more prepared to care for patients with COVID-19. We also observed that the vital-sign data availability followed that which we had previously seen in our wearability study (11), the Nonin capturing 21% less data than the VitalPatch®. The analysis of the amount of data captured per patient was limited to the time they were registered in the system (i.e., ward admission/discharge times were absent from our dataset). Nevertheless, vital-sign data was available to be reviewed by clinical staff, i.e., from the remote nurse bays, for a median of 88.1 [62.5, 94.5]% of the patients' time in the system. The latter, allied to the fact that half of the ward was using at least one wearable device at the peak of the first wave, and that the system was also used during the second and third waves of the pandemic in the UK, are good indicators that such systems are needed to monitor patients in isolating rooms while keeping the hospital staff safe.

### Future Work

The Oxford vHDU AMS has now also been used during the second and third waves of the pandemic in the UK. Therefore, the next steps in evaluating and improving the AMS for the isolation wards, include:

- the analysis of the human factors that might influence the usage of the system in the ward, e.g., *via* focus groups interviews with clinical staff;
- the analysis of patterns in the continuous and intermittent vital-sign data, collected by the vHDU system, vs. COVID-19 patients adverse events (occurring in the isolation ward);
- the ability to combine the continuous HR, RR, and SpO2 data from the wearables with the complementary data from the nurse observations (BP, Temp and level of consciousness), available from the hospital e-obs system (19), to compute the EWS for each patient on the vHDU system;
- the implementation of a notification system for clinical events linked to abnormal physiology (e.g., alerts for low SpO_2_ values for COVID-19 patients), and for technical events, such as missing wearable data (the validation of the scoring and alerting system in a dedicated clinical study is currently being planned);
- and finally, exploring the use of the system outside of the hospital, as some patients could continue to be monitored remotely after they have been discharged home.

## Conclusion

We have developed and deployed a wearables system based on commercial off-the-shelf components, that enables the remote, real-time review of the vital signs of ambulatory COVID-19 patients on a set of individual rooms within the isolation ward of our local hospital. The system was optimised to meet six different requirements which had been established for reliable continuous monitoring of the cardio-respiratory physiology of the patients nursed on this ward. System usage increased when ward occupancy increased during the peak of the first wave of the pandemic in the UK, demonstrating the clinical usefulness of the system.

Beyond the pandemic, we aim to conclude the evaluation of our vHDU wearables system, in which we will evaluate whether the addition of automated alerts to the AMS can help nursing staff identify patient deterioration earlier (between their regular vital-sign observations) in high-dependency or step-down units.

## Availability and Requirements

**Project:** vHDU (virtual High-Dependency Unit).
**Home Page:** https://www.ndcn.ox.ac.uk/research/critical-care-research-group-kadoorie-centre
**Operating System(s):** Android OS for the patient data collection app; the remainder of the system is platform-independent.
**Programming language:** HTML, CSS, JavaScript, PHP, Java, and SQL.
**Other requirements:** Modern web-browser, Android Tablets, VitalPatch® (VitalConnect, USA), WristOx2® 3150 BLE OEM (Nonin, USA) devices.
**Licence:** Proprietary Licence.
**Any restriction to use by non-academics:** licence needed.

## Data Availability Statement

The datasets presented in this article are not readily available because these data requires the permission of the Oxford University Hospitals National Health Service (NHS) Foundation Trust to be used. Requests to access the datasets should be directed to Peter Watkinson, peter.watkinson@ndcn.ox.ac.uk.

## Ethics Statement

The studies involving human participants were reviewed and approved by Oxford University Hospitals National Health Service Foundation Trust. Written informed consent for participation was not required for this study in accordance with the national legislation and the institutional requirements.

## Author Contributions

MS, CR, MP, SV, CA, and LY designed the vHDU software. MS, CR, and MP implemented, tested, and deployed the vHDU software for use in the John Radcliffe Hospital isolation ward. CR, SV, CA, and LY provided training and support to the clinical staff. MS and CR drafted the manuscript. MP and LT helped draught it. LT and PW co-lead the project. All authors reviewed the manuscript and approved it.

## Funding

MS, CR, SV, CA, LY, PW, and LT were funded/supported by the National Institute for Health Research (NIHR) Oxford Biomedical Research Centre (BRC). The views expressed are those of the authors and not necessarily those of the NHS, the NIHR or the Department of Health. MP was funded by a Drayson Research Fellowship.

## Conflict of Interest

LT and PW reports significant grants from the National Institute of Health Research (NIHR), UK and the NIHR Biomedical Research Centre, Oxford, during the conduct of the study; modest grants and personal fees from Sensyne Health, outside the submitted work and hold share options in the company. LT works part-time for Sensyne Health. PW was Chief medical Officer for Sensyne Health until March 2020. The remaining authors declare that the research was conducted in the absence of any commercial or financial relationships that could be construed as a potential conflict of interest.

## Publisher's Note

All claims expressed in this article are solely those of the authors and do not necessarily represent those of their affiliated organizations, or those of the publisher, the editors and the reviewers. Any product that may be evaluated in this article, or claim that may be made by its manufacturer, is not guaranteed or endorsed by the publisher.
